# Copper-catalyzed yne-allylic substitutions: concept and recent developments

**DOI:** 10.3762/bjoc.20.232

**Published:** 2024-10-31

**Authors:** Shuang Yang, Xinqiang Fang

**Affiliations:** 1 State Key Laboratory of Structural Chemistry, and Key Laboratory of Coal to Ethylene Glycol and Its Related Technology, Center for Excellence in Molecular Synthesis, Fujian Institute of Research on the Structure of Matter, University of Chinese Academy of Sciences, Fuzhou 350100, China

**Keywords:** copper-catalysis, copper vinyl allenylidene intermediate, 1,3-enyne, 1,4-enyne, yne-allylic substitution

## Abstract

The catalytic (asymmetric) allylation and propargylation have been established as powerful strategies allowing access to enantioenriched α-chiral alkenes and alkynes. In this context, combining allylic and propargylic substitutions offers new opportunities to expand the scope of transition metal-catalyzed substitution reactions. Since its discovery in 2022, copper-catalyzed yne-allylic substitution has undergone rapid development and significant progress has been made using the key copper vinyl allenylidene intermediates. This review summarizes the developments and illustrates the influences of copper salt, ligand, and substitution pattern of the substrate on the regioselectivity and stereoselectivity.

## Introduction

**Scheme 1:**
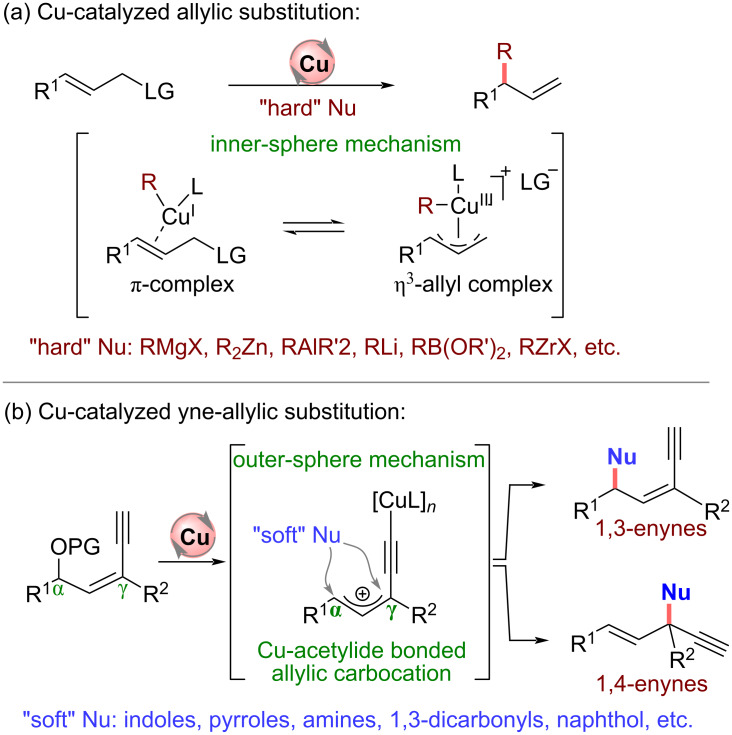
Copper-catalyzed allylic and yne-allylic substitution.

Copper is earth-abundant, inexpensive, relatively stable, and low toxic. Copper-catalyzed asymmetric allylic [1–22] and propargylic [23–32] substitutions are effective strategies for constructing new C–C and C–heteroatom bonds vicinal to alkenyl or alkynyl groups, which are highly valuable for downstream synthesis. At present, unstabilized nucleophiles [33–51] are commonly used in Cu-catalyzed allylic substitutions because of the inner-sphere mechanism and relatively harsh reaction conditions such as anhydrous, anaerobic, and low temperatures are usually required (Scheme 1a). Therefore, using stabilized nucleophiles in Cu-catalyzed allylic substitutions is a tremendous challenge. On the other hand, since the pioneering work of van Maarseveen [52] and Nishibayashi [53] groups in 2008, Cu-catalyzed asymmetric propargylic substitutions have made significant progress [54–60]. The protocol allows the use of stabilized nucleophiles via the outer-sphere mechanism, and the copper allenylidene intermediate formed by copper and terminal alkyne is the active species in the reactions. In this regard, merging the unique feature of Cu-catalyzed propargylic substitution with allylic substitution is a feasible solution to the challenge, which will represent a new sort of substitution reaction.

**Scheme 2:**
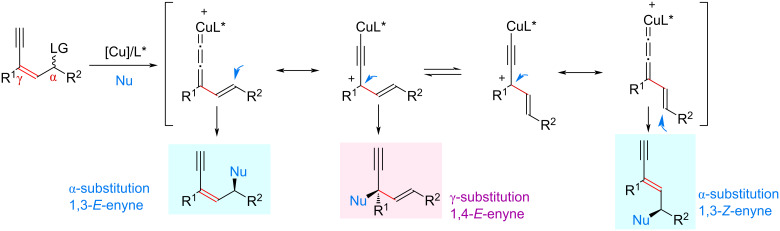
Challenges in achieving highly selective yne-allylic substitution.

From 2022, the Cu-catalyzed yne-allylic substitution has emerged as a new and robust approach to achieve formal allylic substitution using stabilized nucleophiles. The copper acetylide-bonded allylic cation with copper vinyl allenylidene species as its resonance structure is key for the process, which can achieve the outer-sphere attack of nucleophiles (Scheme 1b). However, to achieve a highly selective yne-allylic substitution, a range of challenges must be addressed. First, how to achieve the regioselectivity under the coexistence of alkenyl and alkynyl units; second, how to realize the enantioselectivity control that is remote from the catalytic center; finally, the selectivity affording *E*-enyne and *Z*-enyne product is also an issue to be addressed, and possible side reactions need to be suppressed (Scheme 2).

In this review, we summarize the recent development of copper-catalyzed yne-allylic substitutions. It is worth noting that when we were preparing this review, another review on copper-catalyzed asymmetric propargylic substitution including some yne-allylic substitutions was reported by Lin et al. [61]. We hope that this review can provide more guidance for the use of new nucleophiles and the development of new reaction modes related to yne-allylic substitutions.

## Review

### Copper-catalyzed yne-allylic substitutions affording 1,3- and 1,4-enynes

**Scheme 3:**
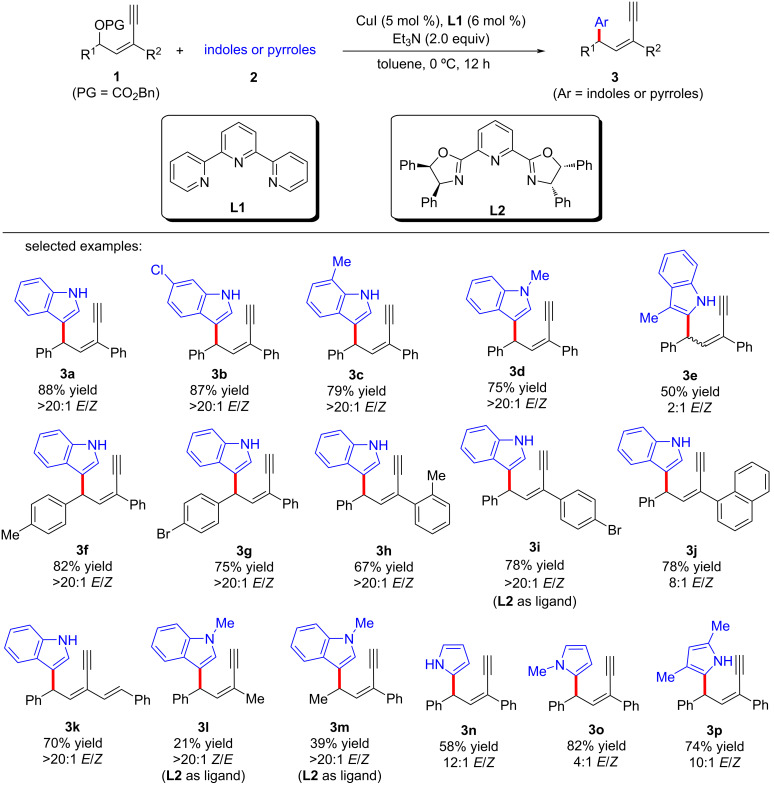
Yne-allylic substitutions using indoles and pyroles.

**Scheme 4:**
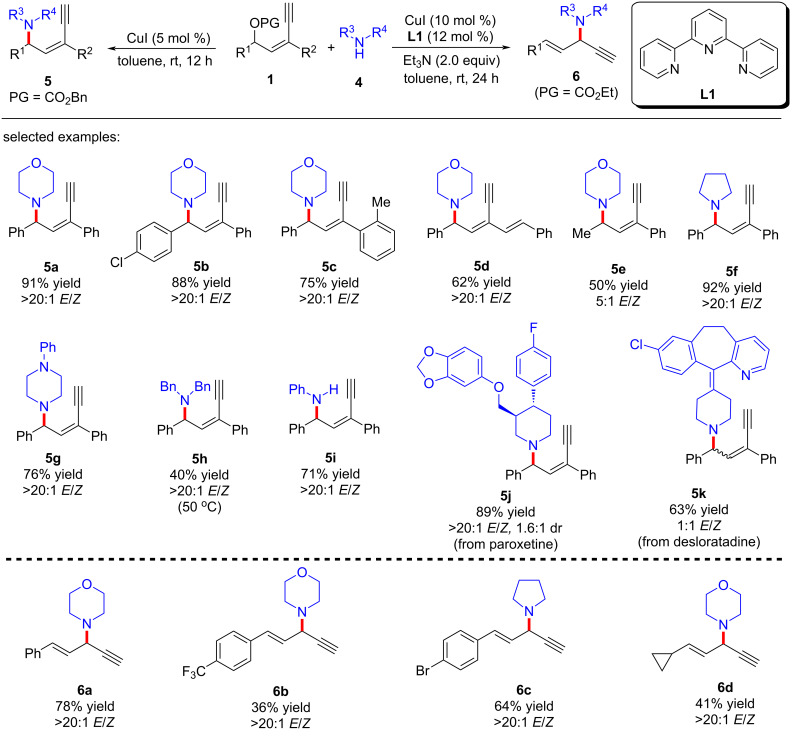
Yne-allylic substitutions using amines.

**Scheme 5:**
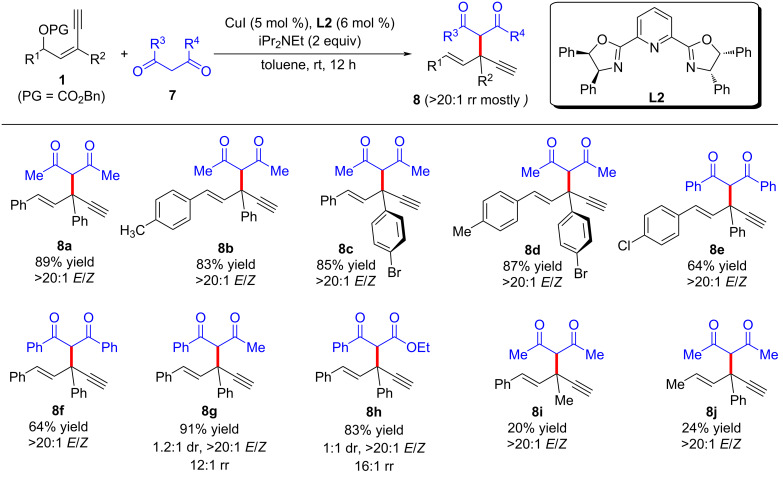
Yne-allylic substitution using 1,3-dicarbonyls.

**Scheme 6:**
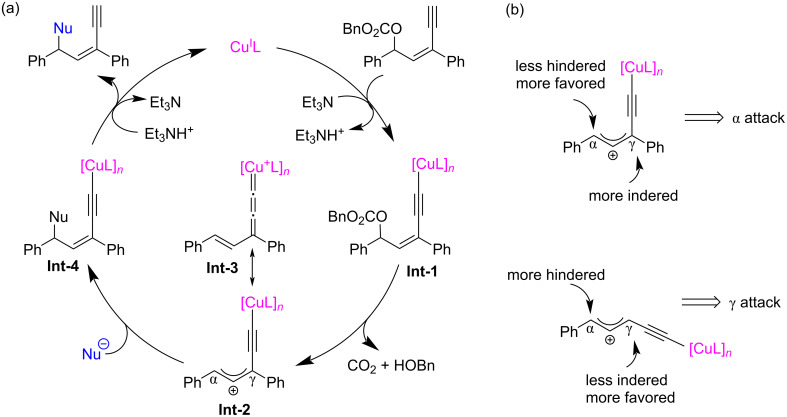
Postulated mechanism via copper acetylide-bonded allylic cation.

In 2022, Fang et al. [62] realized the copper-catalyzed yne-allylic substitution involving stabilized “soft” nucleophiles for the first time. Indoles and pyrroles with various substituents were found to be suitable for the reaction, delivering 1,3-enynes with medium to high yields and excellent regioselectivities (Scheme 3, **3a**–**e**, **3n**–**p**). Interestingly, when the 3-position of indole was blocked by a methyl group, the 2-position of indole underwent nucleophilic attack with an *E*/*Z* ratio of 2:1 (Scheme 3, **3e**). Carbonates with aryl or styryl residue can undergo the reaction smoothly (Scheme 3, **3f**–**k**), but alkyl-substituted substrates showed low yields (Scheme 3, **3l** and **3m**). Moreover, secondary amines with various substituents, acyclic amines, primary amines, or even the amine moieties in drug molecules were all suitable nucleophiles (Scheme 4, **5a**–**k**). When R^2^ is an H atom, the reactions occur at the γ-position, resulting in the formation of 1,4-enynes (Scheme 4, **6a**–**d**). Acyclic 1,3-dicarbonyls could also react with yne-allylic carbonates **1** at the γ-position because of the possible chelation interaction between the enolate derived from acyclic 1,3-dicarbonyl compounds and copper (Scheme 5, **8a**–**j**). Detailed control experiments indicate that the terminal alkyne moiety is critical and the reaction proceeds through an S_N_1 mechanism. An outer-sphere nucleophilic attack through copper acetylide-bonded allylic cation as the key intermediate is proposed (Scheme 6a). It is worth noting that the nucleophilic attack favors a less sterically hindered site. Therefore, disubstituted alkene moiety prefers γ-attack while trisubstituted alkene moiety is inclined to α-attack (Scheme 6b).

**Scheme 7:**
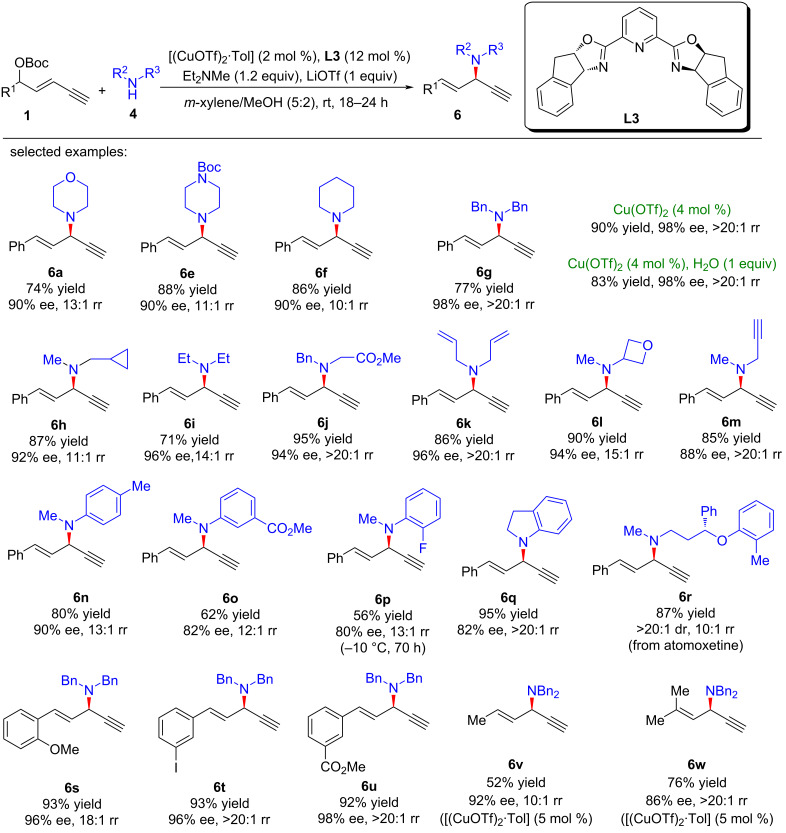
Amine-participated asymmetric yne-allylic substitution.

**Scheme 8:**
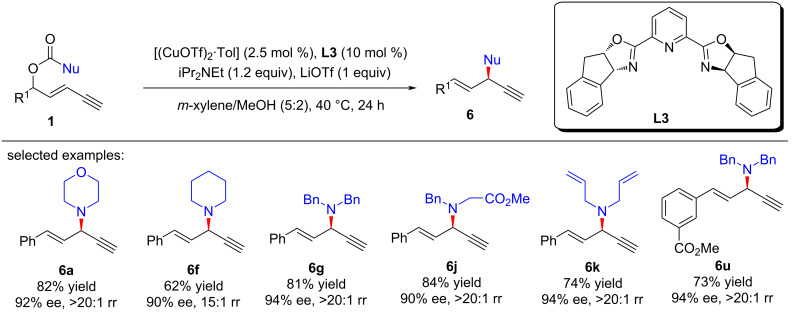
Asymmetric decarboxylative yne-allylic substitution.

**Scheme 9:**
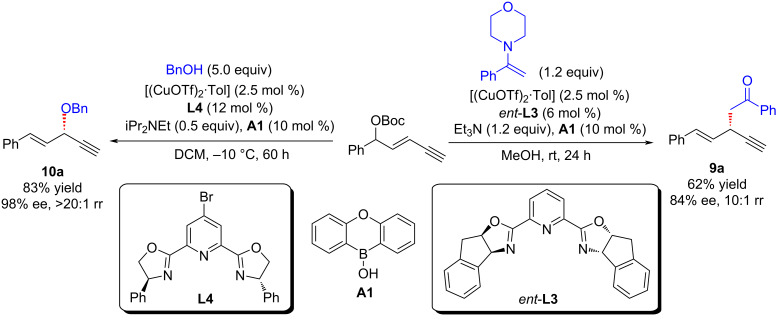
Asymmetric yne-allylic alkoxylation and alkylation.

**Scheme 10:**
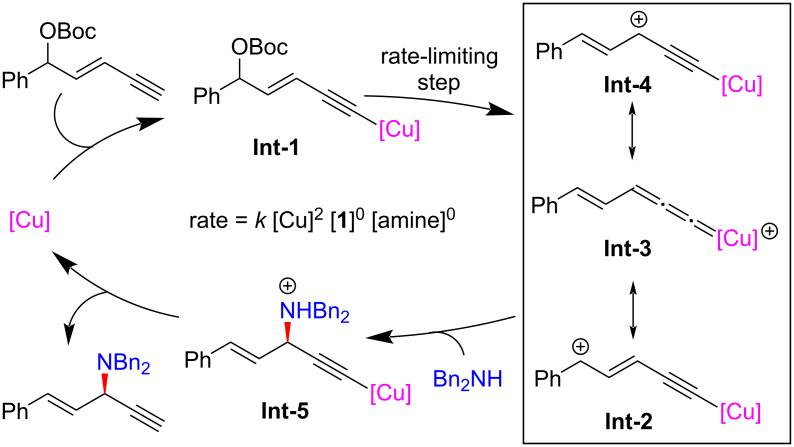
Proposed mechanism for Cu(I) system.

Lin and He et al. [63–64] reported the first amine-mediated highly enantioselective copper-catalyzed asymmetric yne-allylic substitution, affording 1,4-enynes with up to 98% ee and >20:1 rr. A series of secondary amines can react smoothly and achieve good enantioselectivities and regioselectivities (Scheme 7, **6a**–**w**). Interestingly, both Cu(I) and Cu(II) can promote the reaction and the reaction is not sensitive to water (Scheme 7, **6g**). The intramolecular decarboxylative yne-allylic substitution can also be achieved (Scheme 8, **6a**–**u**). *O*-Nucleophiles and *C*-nucleophiles are all suitable reactants, yielding alkoxylation and alkylation products with the assistance of Lewis acid as co-catalyst (Scheme 9). Starting from four different racemic substrates, the same product **6g** with 96% ee was obtained under standard conditions. This indicates that the reactions proceed through the same transition state and the stereocenter of the product is controlled by the catalyst. A single crystal of Cu(I) was investigated by X-ray and proved to be the dicopper complex, while the Cu(II) catalyst was revealed as mononuclear copper coordinated with two ligands. Further kinetic isotope experiments and nonlinear relationship studies for the Cu(I) system indicate that it is not the formation of alkynyl copper intermediate but the formation of active copper vinyl allenylidene intermediate is the rate-limiting step (Scheme 10).

**Scheme 11:**
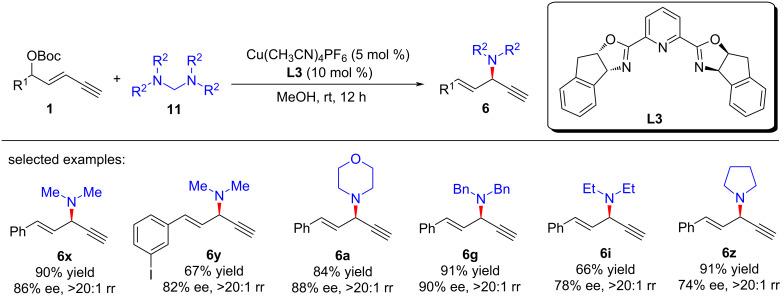
Asymmetric yne-allylic dialkylamination.

**Scheme 12:**
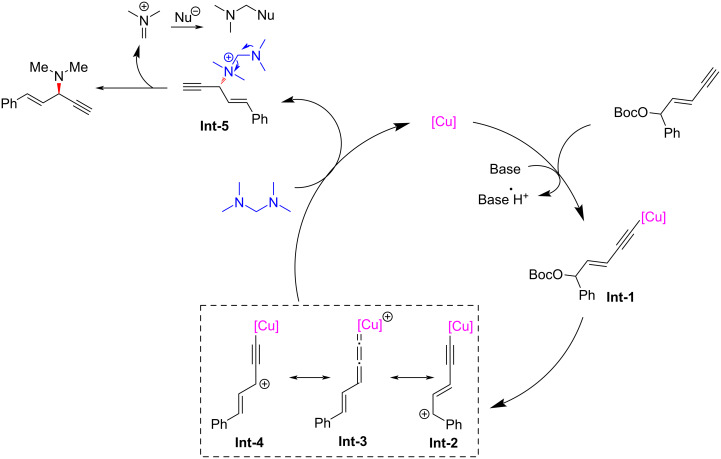
Proposed mechanism of yne-allylic dialkylamination.

Due to the gaseous nature of dimethylamine at room temperature, it needs to be stored in special solvents, which further limits the preparation of the related compounds. He et al. [65] used tetramethyldiaminomethane as a suitable surrogate of dimethylamine to achieve the asymmetric dimethylamination of yne-allylic esters, providing an efficient and convenient pathway for the synthesis of enantioenriched 1,4-enynes with dimethylamine moiety (Scheme 11, **6x** and **6y**). In addition to tetramethylenediaminomethane, other tetraalkyldiaminomethanes can also be used as supplants of dialkylamines, leading to products with dialkylamine units with high yields and enantioselectivities (Scheme 11, **6a**, **6g**, **6i** and **6z**). Further control experiments and DFT calculations show that during the catalytic process, tertiary amine directly participates as a nucleophilic reagent to give the ammonium salt, which then releases dimethylaminium to provide the final product (Scheme 12).

**Scheme 13:**
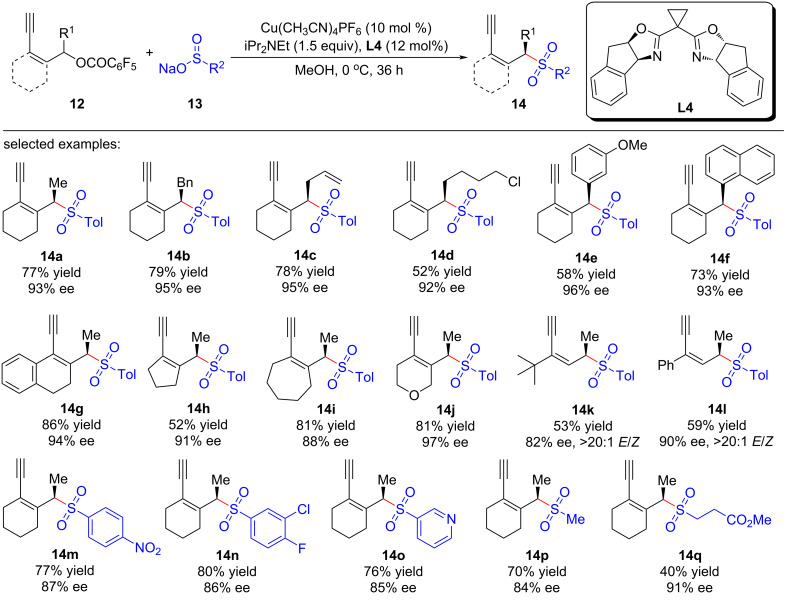
Asymmetric yne-allylic sulfonylation.

**Scheme 14:**
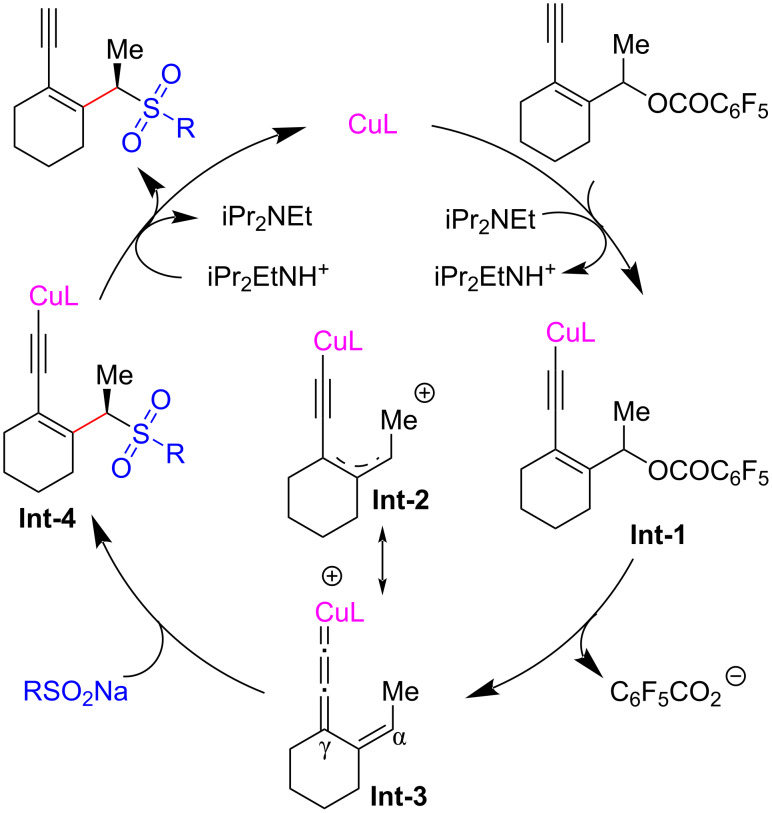
Proposed mechanism of yne-allylic sulfonylation.

Chiral allylic sulfone compounds can be easily transformed into a series of useful molecules, enabling them an important backbone in organic synthesis. Lin et al. [66] used sodium sulfinates as the nucleophiles to realize the asymmetric sulfonylation of yne-allylic esters. The reaction can be carried out under mild conditions with good to excellent regio-, stereo-, and enantioselectivities, resulting in a series of chiral yne-allylic sulfone compounds with different substituents (Scheme 13, **14a**–**q**). Due to the high steric hindrance of the γ-site, nucleophilic substitutions preferentially occur at the α-site. Through subsequent control experiments, they demonstrated that the terminal alkyne unit is crucial for the process and the reactions using different isomers all proceed via the same intermediate. Nonlinear relationship experiments proved that the active catalyst is a mono-copper complex containing one ligand. A catalytic cycle is proposed in which copper vinyl allenylidene is the key intermediate during the process (Scheme 14).

**Scheme 15:**
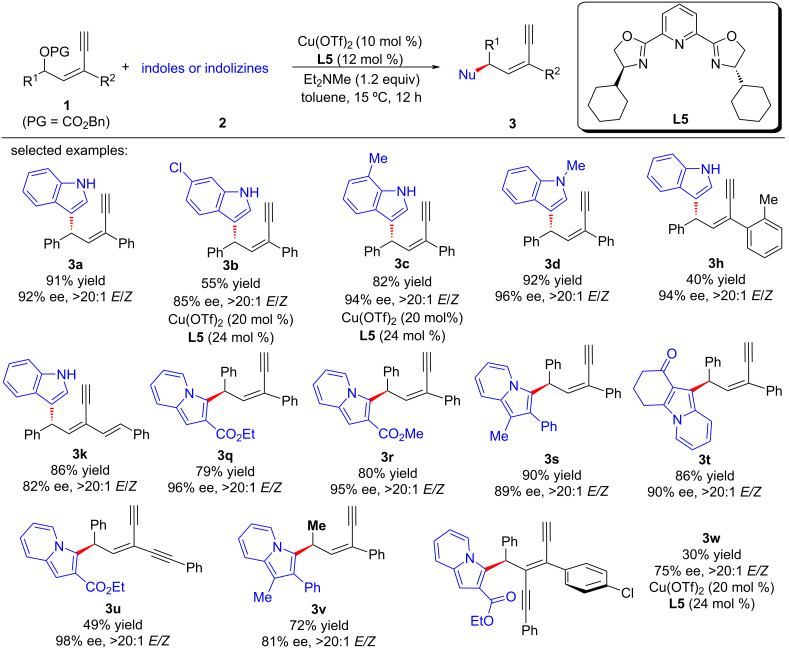
Aymmetric yne-allylic substitutions using indoles and indolizines.

**Scheme 16:**
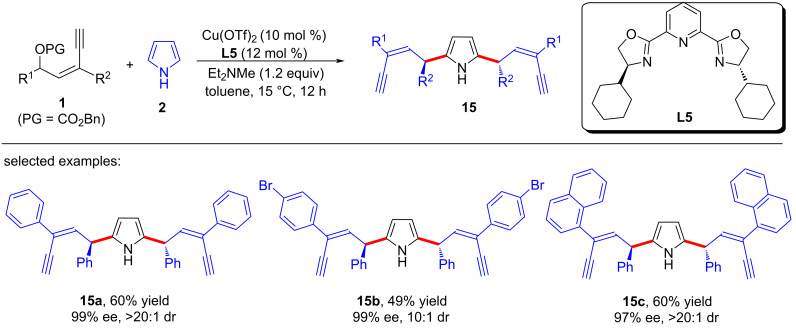
Double yne-allylic substitutions using pyrrole.

**Scheme 17:**
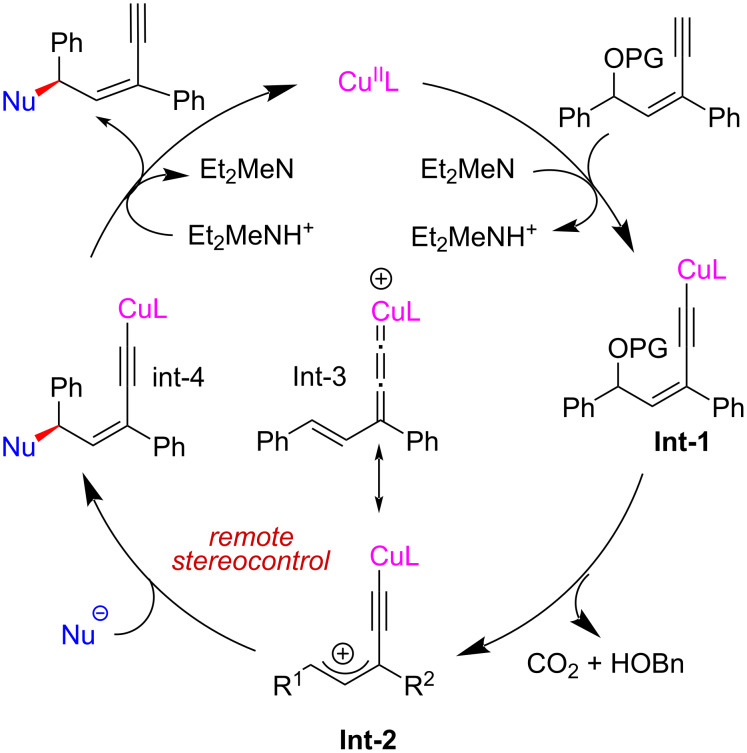
Proposed mechanism of yne-allylic substitution using electron-rich arenes.

Recently, Fang et al. [67] used electron-rich arenes as the nucleophiles to achieve remote enantioselective control of yne-allylic substitutions. It is worth noting that when indoles or indolizines were used, the reactions yielded mono yne-allylic substituted products (Scheme 15, **3a**–**w**), but while pyrroles were used as nucleophiles, double yne-allylic substituted products can be obtained with high dr and ee values (Scheme 16, **15a**–**c**). They also demonstrated the importance of terminal alkyne through control experiments and confirmed that a copper–ligand monomer complex exists in the mechanism through nonlinear relationship experiments and kinetic studies (Scheme 17).

**Scheme 18:**
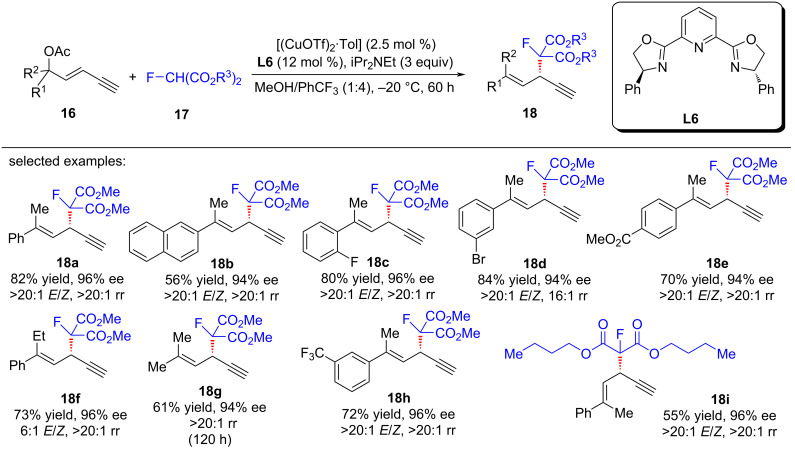
Aymmetric yne-allylic monofluoroalkylations.

**Scheme 19:**
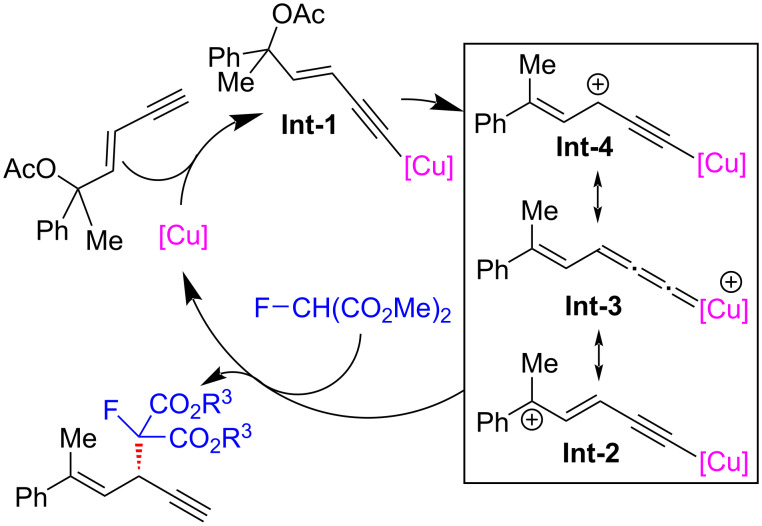
Proposed mechanism.

He et al. [68] developed the regio- and enantioselective monofluoroalkylation of yne-allylic esters using fluorinated malonates as the starting materials, giving rise to a series of differently substituted 1,4-enynes with monofluoroalkyl units in high yields and ee values (Scheme 18, **18a**–**i**). The reaction effectively overcomes the drawbacks of low activity of tertiary carbon nucleophiles and instability of fluorinated compounds. Preliminary mechanistic studies showed that the reaction exhibits a negative nonlinear effect, while the kinetic experiments indicate that a mono-copper catalyst might be involved in the rate-limiting step. They speculated that the observed nonlinear effect might arise from the existence of both the inactive homo-dimer of ligands and active mono-copper species in the enantio-determining step (Scheme 19).

**Scheme 20:**
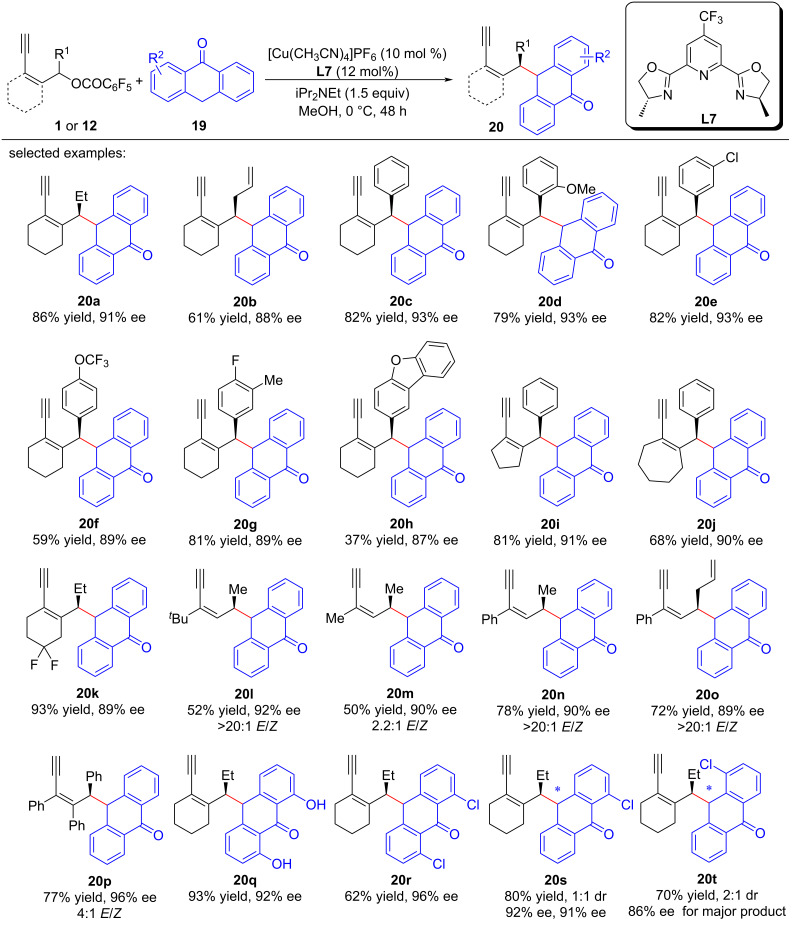
Aymmetric yne-allylic substitution of yne-allylic esters with anthrones.

Lin et al. [69] achieved, for the first time, the asymmetric yne-allylic substitution using anthrones as the substrates by using a *p*-CF_3_ substituted Pybox ligand, yielding 1,3-enynes containing anthrone units with high regioselectivities and stereoselectivities (Scheme 20). The reaction tolerated various substituents in the yne-allylic esters (Scheme 20, **20a**–**h**), and showed good chiral induction effects for other multiring substrates (Scheme 20, **20i**–**k**) and linear substrates as well (Scheme 20, **20l**–**o**). A disubstituted yne-allylic ester was also a suitable substrate for the reaction (Scheme 20, **20p**). In addition, the presence of substituents such as hydroxy or chlorine groups on the anthrones had no impact on the reaction (Scheme 20, **20q**–**t**).

**Scheme 21:**
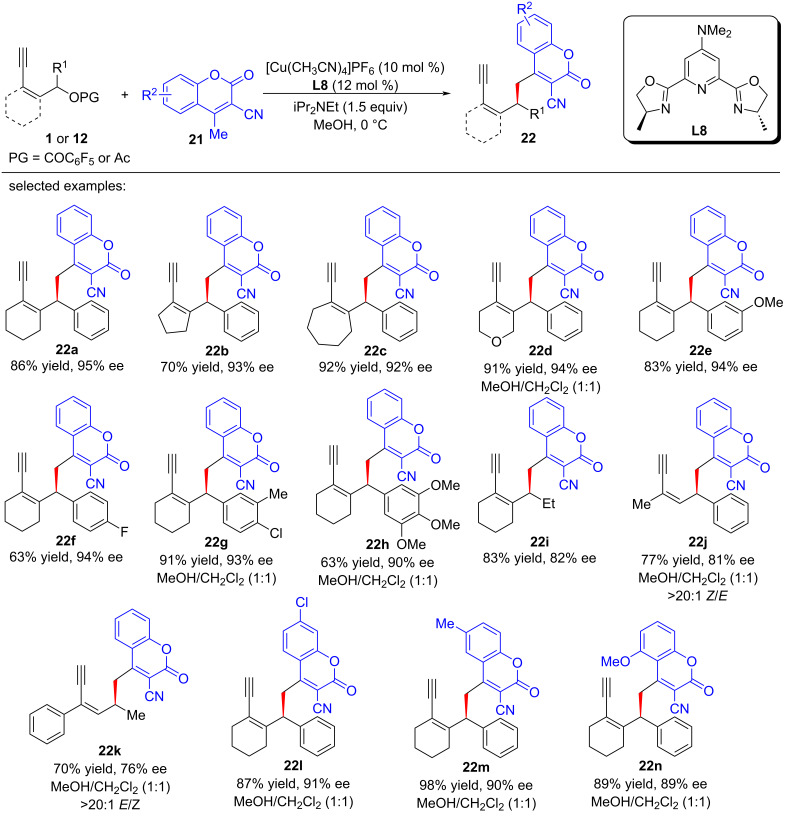
Aymmetric yne-allylic substitution of yne-allylic esters with coumarins.

**Scheme 22:**
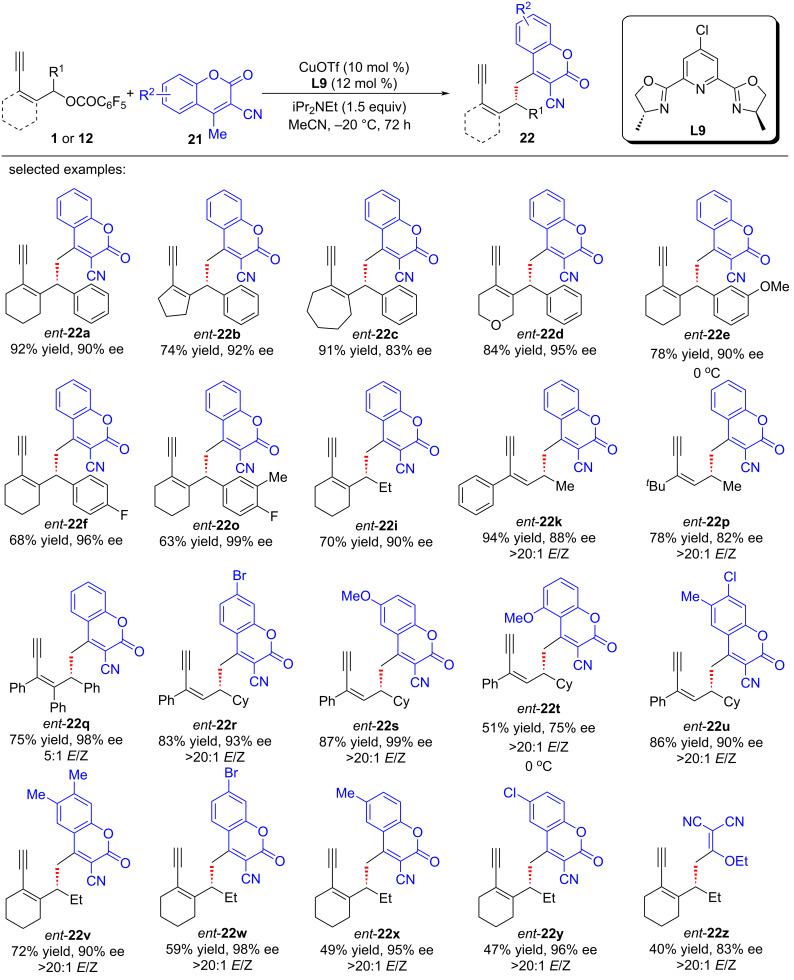
Aymmetric yne-allylic substitution of with coumarins by Lin.

Chiral coumarins, renowned for their bioactive properties, form the cornerstone of numerous natural products and pharmaceutical prospects. Given their significance, the pursuit of efficient and direct synthetic routes to access functionalized chiral coumarins has garnered substantial interest. While the literature is abundant with reports on coumarin-based propargylic and allylic substitutions, the realm of yne-allylic substitution remains relatively unexplored. This scarcity of reports stems primarily from the inherent challenges posed by the presence of two unsaturated bonds in the substrates, which often complicates the control of regioselectivity. Additionally, the competition among various nucleophilic reagents further hampers the reactivity of coumarins. Xu, Peng and Feng et al. [70] introduce a groundbreaking dual remote enantioselective copper-catalyzed yne-allylic substitution methodology tailored specifically for coumarins (Scheme 21). This innovative approach facilitates the precise and highly regioselective construction of an array of novel chiral coumarin derivatives, characterized by their exceptional synthetic efficiency and remarkable tolerance towards a broad spectrum of functional groups (Scheme 21, **22a**–**n**).

**Scheme 23:**
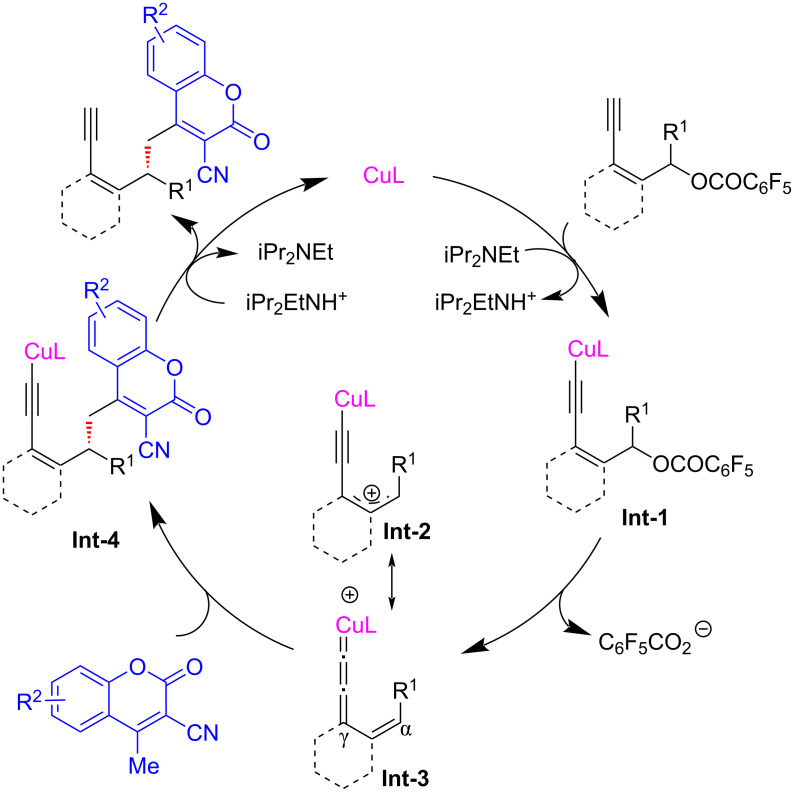
Proposed mechanism.

Almost at the same time, Lin et al. [71] also devised a copper-catalyzed protocol for the vinylogous yne-allylic substitution utilizing coumarins as substrates (Scheme 22). The methodology stands out for its exceptional regio and enantioselectivities, while employing readily available starting materials and mild reaction conditions, showcasing the robustness of the method to access structurally diverse chiral coumarin derivatives, which are of great interest in the fields of medicinal chemistry and synthetic organic chemistry (Scheme 22, **22a**–**y**). Moreover, the utilization of 2-(1-ethoxyethylidene)malononitrile as a γ-nucleophile yielded the desired product **22z** with a moderate 40% yield, but impressively high enantiomeric excess of 83%. To further validate the mechanistic pathway of the reaction, the authors conducted both radical trapping experiments and controlled experiments. These investigations conclusively demonstrated that the reaction did not proceed via a radical mechanism and the presence of terminal alkynes was found to be crucial for the smooth progression of the reaction, which suggested that the reaction proceeded through the same copper vinyl allenylidene intermediate (Scheme 23).

### Yne-allylic substitutions through dearomatization and rearomatization

**Scheme 24:**
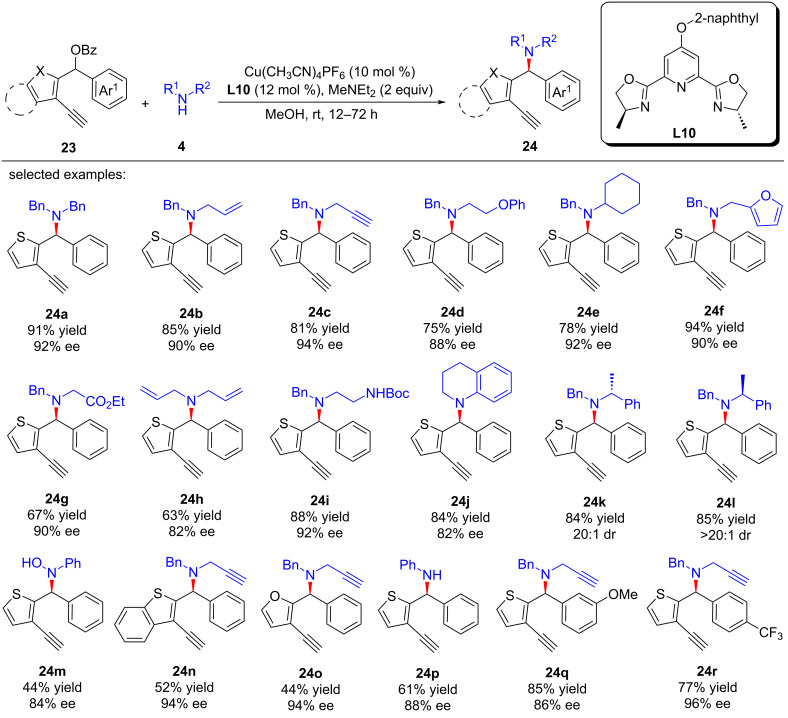
Amination by alkynylcopper driven dearomatization and rearomatization.

**Scheme 25:**
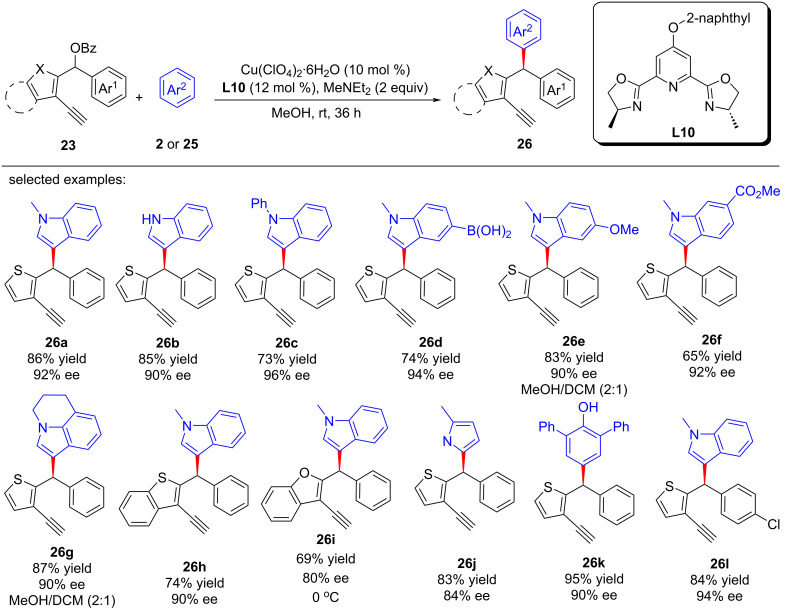
Arylation by alkynylcopper driven dearomatization and rearomatization.

**Scheme 26:**
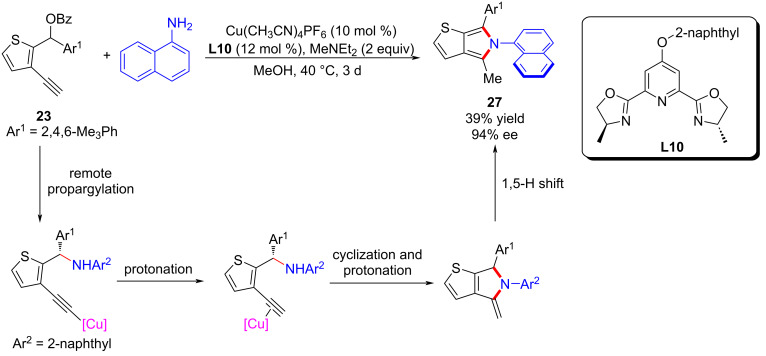
Remote substitution/cyclization/1,5-H shift process.

**Scheme 27:**
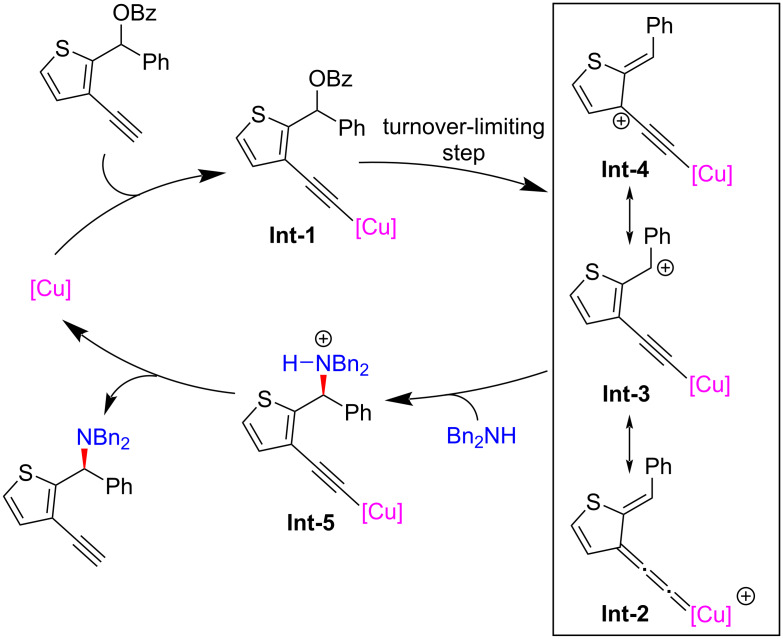
Proposed mechanism.

In 2023, Lin and He et al. [72] achieved the challenging dearomatization of heteroarenes through d-orbital electron of the transition-metal center and thus completed the asymmetric substitutions with remote stereoselective control induced by alkynylcopper. A newly electron-rich ligand was developed to enhance the back donation of d-orbital electron of copper, thereby achieving dearomatization and rearomatization with excellent yields and enantioselectivities. A series of synthesized useful diarylmethyl (Scheme 24, **24a**–**r**) and triarylmethyl (Scheme 25, **26a**–**l**) structures were obtained. Moreover, they also achieved the construction of C–N axis chirality through remote substitution/cyclization/1,5-H shift process (Scheme 26). The control experiments confirmed that the reaction requires the joint participation of copper and terminal alkyne, and the radical-capture experiment also ruled out a radical-involved mechanism. In addition, a positive nonlinear relationship between the product and ligand indicated that the dinuclear copper is the active catalyst, which was also proved by single crystal X-ray analysis. However, kinetic experiments showed the reaction is first-order on copper, implying that the dinuclear copper complex is the precursor of the active monocopper species which is involved in the turnover-limiting step (Scheme 27).

**Scheme 28:**
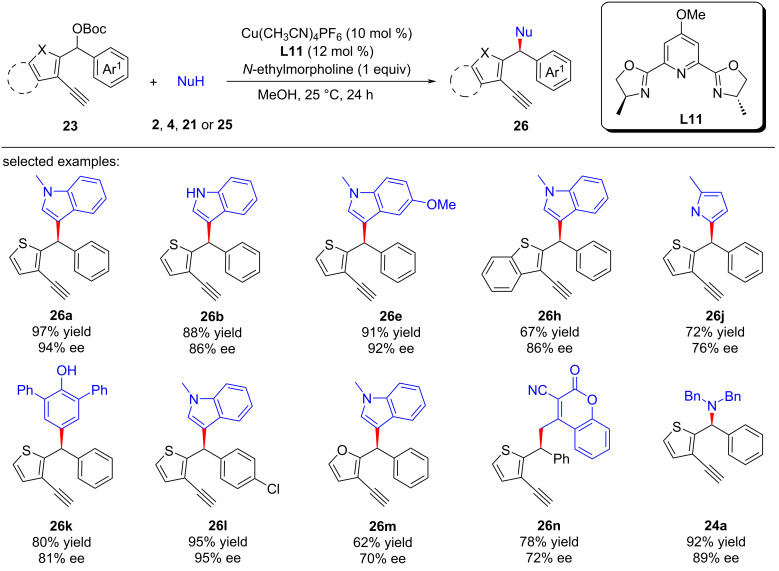
Arylation or amination by alkynylcopper driven dearomatization and rearomatization.

Subsequently, Zhu and Xu et al. [73] also achieved the distal enantioselective heteroarylation of yne-thiophene carbonates by applying a simpler *p*-OMe substituted pybox ligand (Scheme 28, **26a**–**m**). The thiophene unit of the carbonate could also be replaced by benzothiophene (Scheme 28, **26h**) or furan (Scheme 28, **26m**), and pyrrole (Scheme 28, **26j**), phenol (Scheme 28, **26k**); coumarin derivative (Scheme 28, **26n**), and dibenzylamine (Scheme 28, **24a**) could also undergo the reactions smoothly as nucleophilic reagents, producing the products with high enantioselectivities. They also conducted radical-trapping experiments and confirmed that the reaction does not involve a radical intermediate. Unlike the previous yne-allyllic substitutions, this reaction could be carried out without the presence of terminal alkyne, although no ee value was obtained. Therefore, they speculated that the direct substitution at the benzyl position is the key to causing the side reaction that affected the enantioselectivity.

**Scheme 29:**
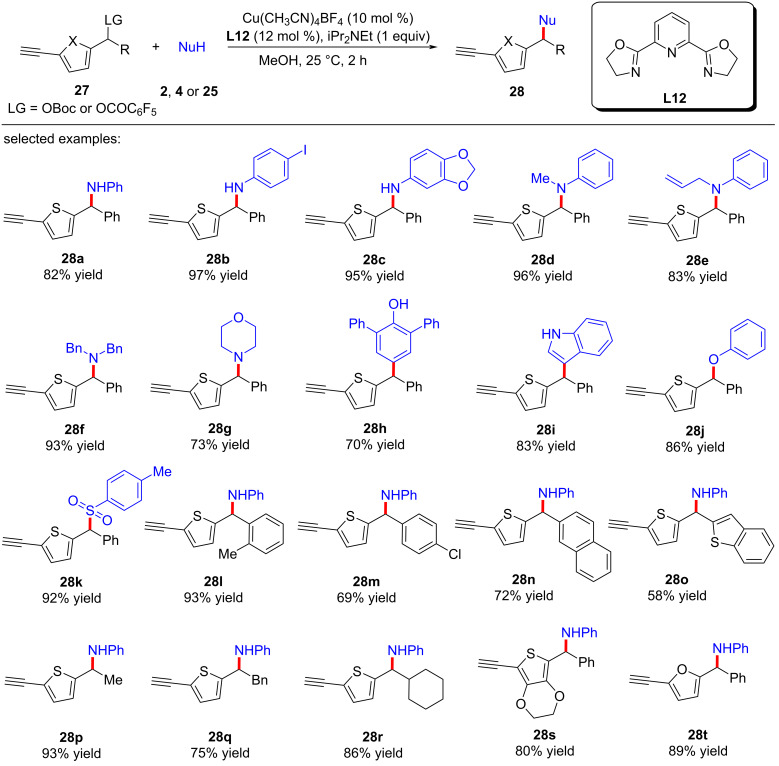
Remote nucleophilic substitution of 5-ethynylthiophene esters.

**Scheme 30:**
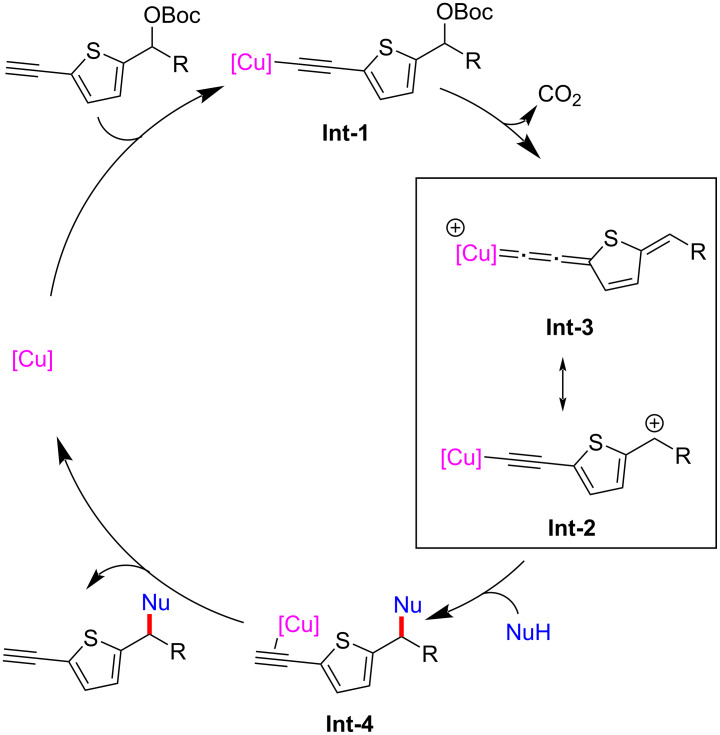
Proposed mechanism.

Recently, Zhu and Xu et al. [74] achieved the η-nucleophilic substitutions of 5-ethynylthiophene esters. A series of C-, N-, O-, and S-nucleophiles could react smoothly to obtain various thiophene derivatives with different functional groups (Scheme 29, **28a**–**t**). Control experiments showed that terminal alkyne and copper catalysts are crucial for the smooth progress of the reaction. Therefore, they believe that the dearomatization caused by copper vinyl allenylidene intermediate remains a key step in the reaction (Scheme 30).

### Copper-catalyzed yne-allylic substitution–annulation reactions

**Scheme 31:**
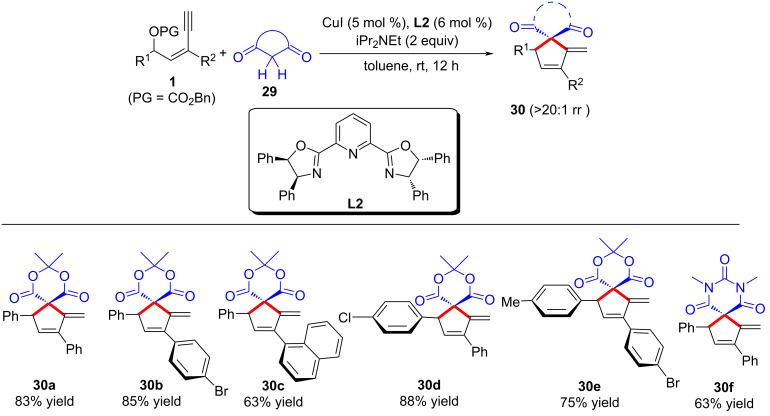
[4 + 1] annulation of yne-allylic esters and cyclic 1,3-dicarbonyls.

In the pioneering report [62], Fang et al. found that when cyclic 1,3-dicarbonyls such as Meldrum’s acid and 1,3-dimethylbarbituric acid were used as the nucleophiles, the yne-allylic substitution products underwent further intramolecular cyclizations to give the spiro-cyclic products in high yields (Scheme 31, **30a**–**f**). The generation of products likely begins with an α-attack on the yne-allylic cation intermediate, followed by an intramolecular cyclization. The disparity in reactivity could stem from the chelation between acyclic 1,3-dicarbonyl enolates and the copper catalyst, enhancing γ-position attack in an intramolecular manner. Conversely, Meldrum's acid's rigid cyclic structure precludes stable copper-carbonyl interaction, favoring attack from a less hindered site.

**Scheme 32:**
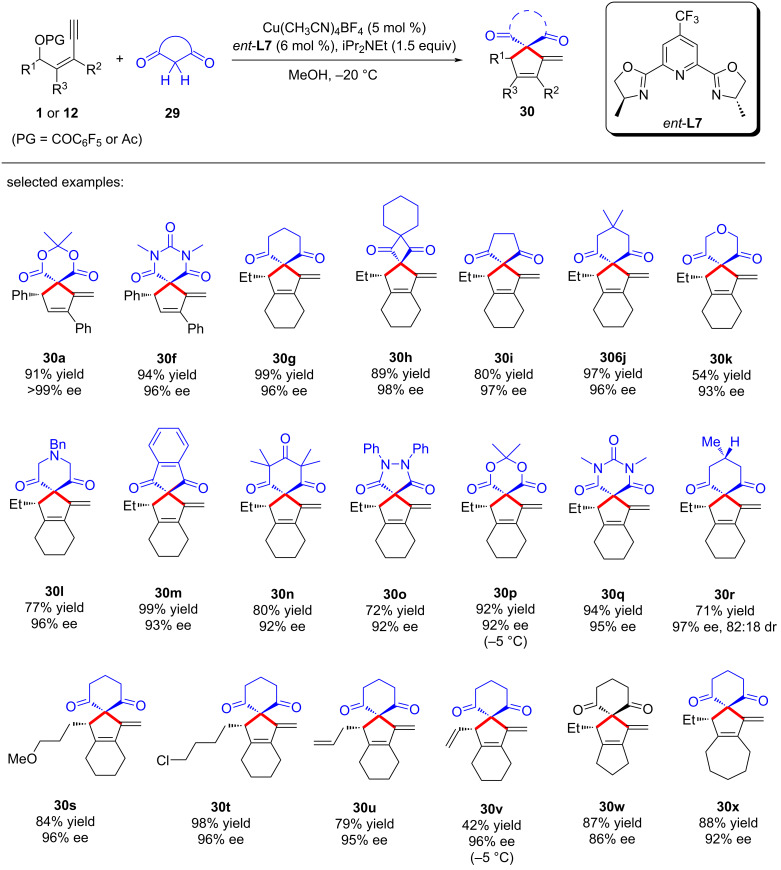
Asymmetric [4 + 1] annulation of yne-allylic esters.

**Scheme 33:**
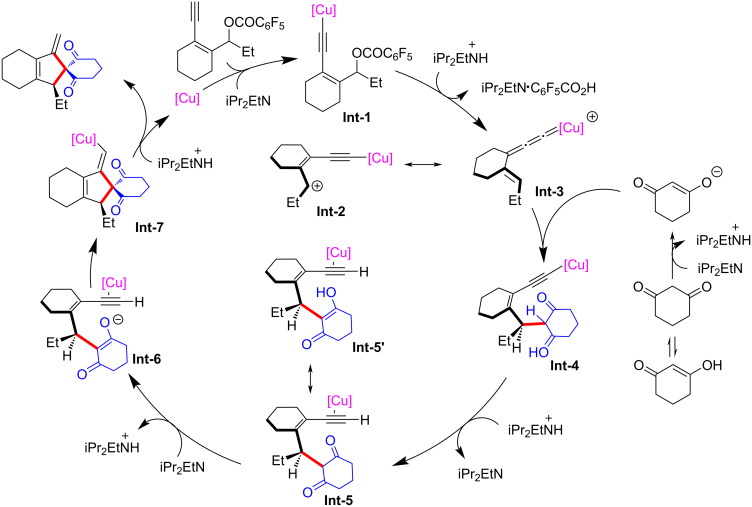
Proposed mechanism.

Later, Qi and Xu et al. [75] achieved highly enantioselective copper-catalyzed [4 + 1] cyclization of yne-allylic esters and cyclic 1,3-dicarbonyls, achieving remote stereoselective control through copper vinyl allenylidene species. A series of differently substituted spiro-cyclic products can be obtained with high yields, regio- and stereoselectivities (Scheme 32, **30a**–**x**). Preliminary mechanistic studies indicated that the reaction first undergoes a substitution at the α-position of yne-allylic ester, followed by a Conia-ene cyclization. The absolute configuration of the products is controlled by the ligand, which further confirms the remote stereocontrol of the copper catalyst. They obtained a single crystal of dinuclear copper and confirmed that the catalytic efficiency of the single crystal is consistent with the standard reaction conditions. However, nonlinear effect experiments confirmed that the active catalyst is a mono-copper species, so it is speculated that the dinuclear copper is the precursor of active single copper species (Scheme 33).

**Scheme 34:**
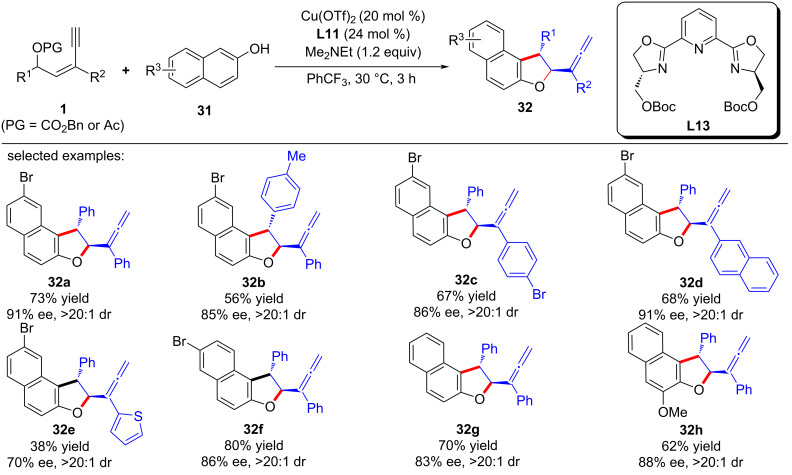
Asymmetric [3 + 2] annulation of yne-allylic esters.

**Scheme 35:**
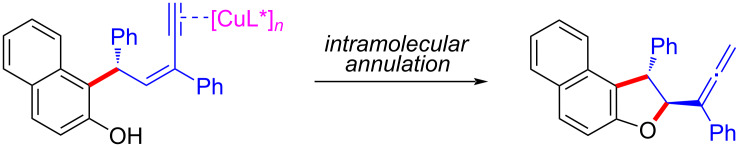
Postulated annulation step.

Fang et al. [67] achieved the first asymmetric [3 + 2] cyclization of yne-allylic esters and 2-naphthalenols, resulting in a range of allenyl dihydronaphthofuran products with high diastereo- and enantioselectivities (Scheme 34, **32a**–**h**). The attempt to separate the intermediate before cyclization failed, indicating that the following annulation and the formation of allene is very fast. It is speculated that in the formation of allene the copper catalyst still plays a key role in activating the alkyne unit (Scheme 35).

**Scheme 36:**
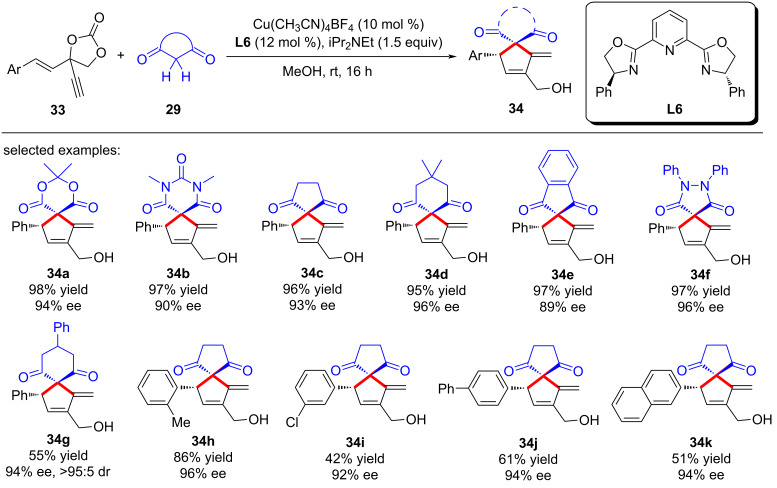
[4 + 1] Annulations of vinyl ethynylethylene carbonates and 1,3-dicarbonyls.

**Scheme 37:**
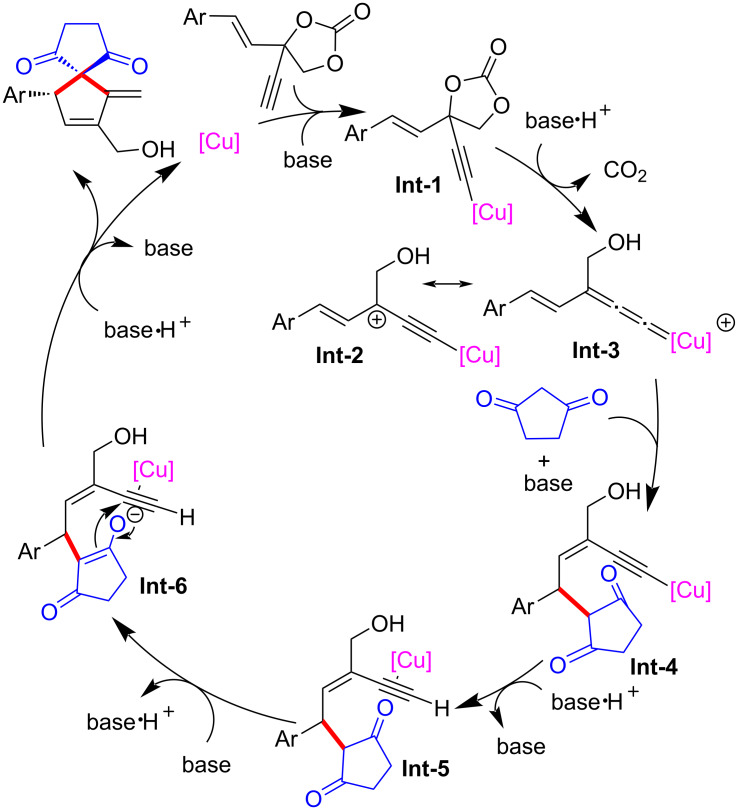
Proposed mechanism.

Han and Huang et al. [76] also achieved the yne-allylic substitution and Conia-ene cyclization process using vinyl ethynylethylene carbonates as the starting materials, thus completing their enantioselective formal [4 + 1] cycloadditions with cyclic 1,3-dicarbonyl compounds (Scheme 36, **34a**–**k**). They speculated that in the reaction mechanism, the key step is the formation of the copper vinyl allenylidene intermediate from vinyl ethynylethylene carbonates and copper catalysts (Scheme 37).

**Scheme 38:**
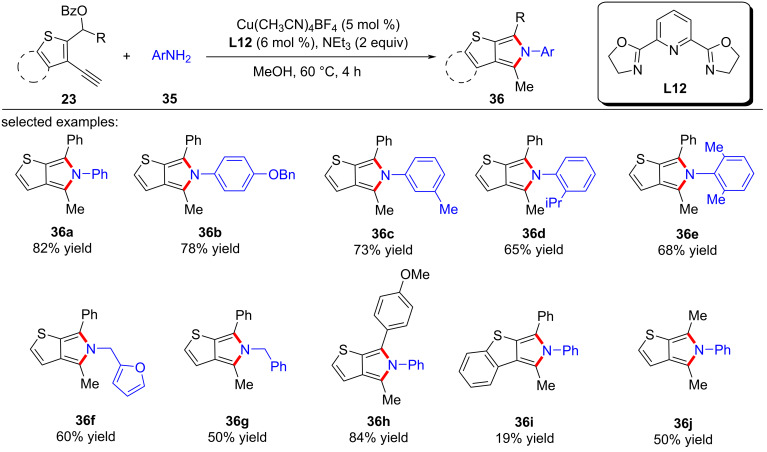
Formal [4 + 1] annulations with amines.

**Scheme 39:**
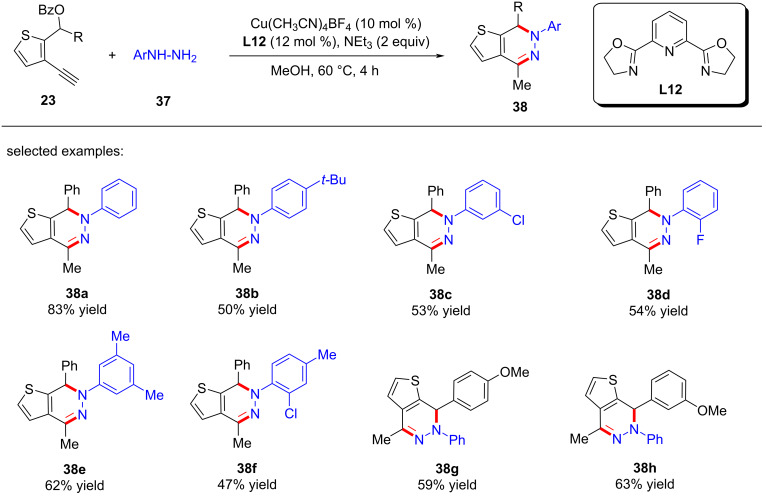
Formal [4 + 2] annulations with hydrazines.

**Scheme 40:**
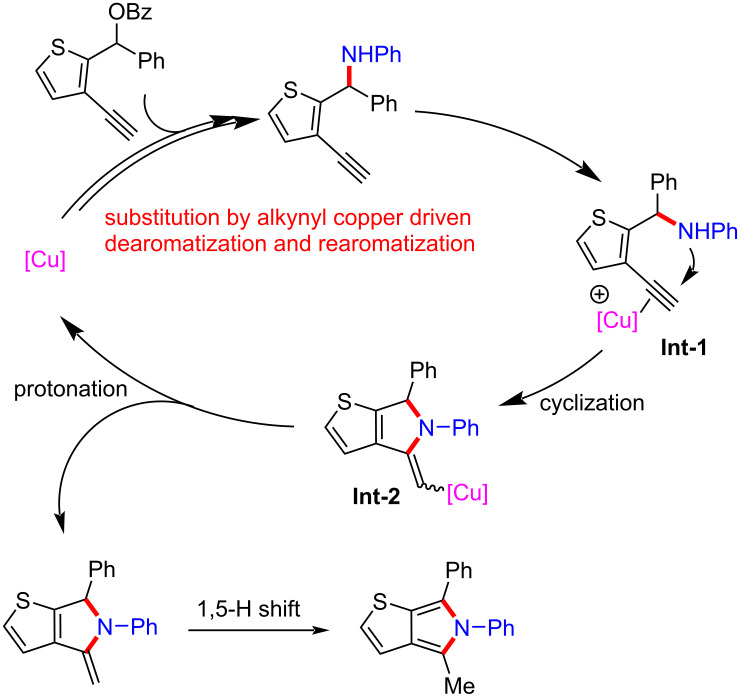
Proposed mechanism.

He et al. [77] completed formal [4 + 1] and [4 + 2] annulations and obtained two types of seldomly studied heterocycles of thieno[2,3-*c*]pyrrole (Scheme 38, **36a**–**j**) and thieno[2,3-*d*]pyridazine (Scheme 39, **38a**–**h**) in high yields. It is worth noting that the formal [4 + 1] and [4 + 2] cyclizations were carried out through substitution by an alkynyl copper-driven dearomatization/rearomatization/cyclization/isomerization process (Scheme 40).

**Scheme 41:**
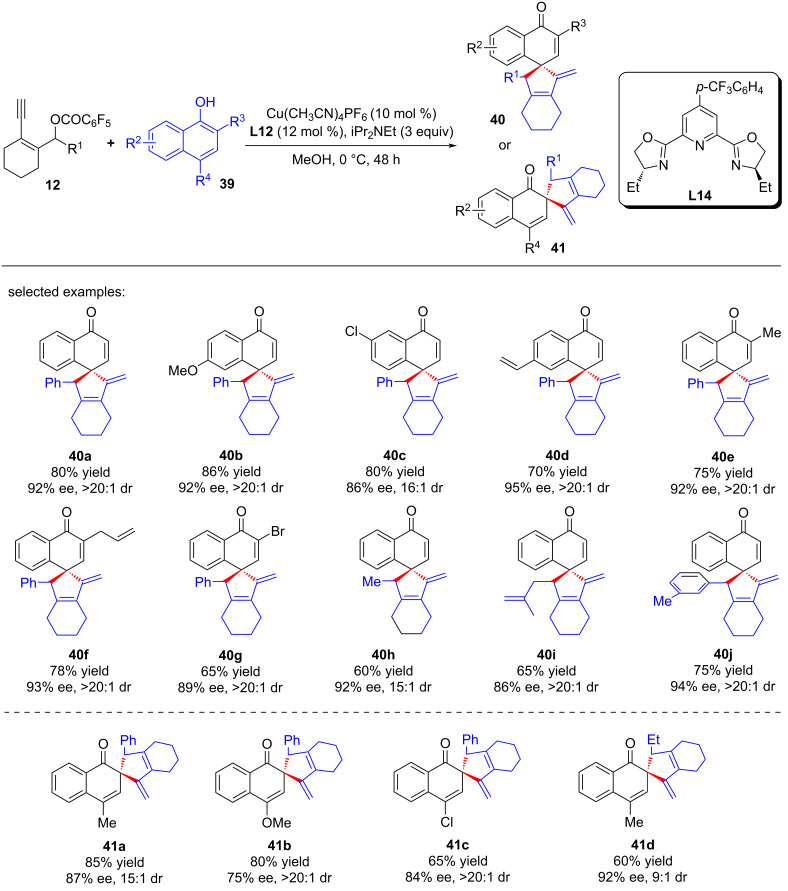
Dearomative annulation of 1-naphthols and yne-allylic esters.

**Scheme 42:**
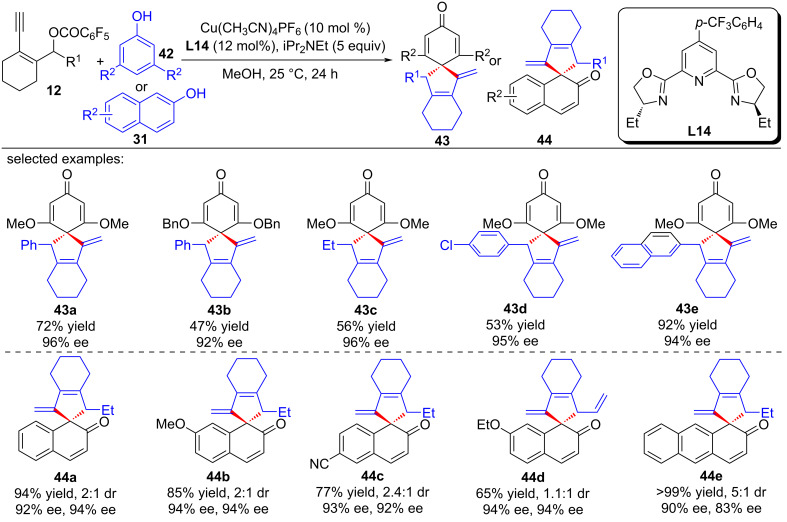
Dearomative annulation of phenols or 2-naphthols and yne-allylic esters.

**Scheme 43:**
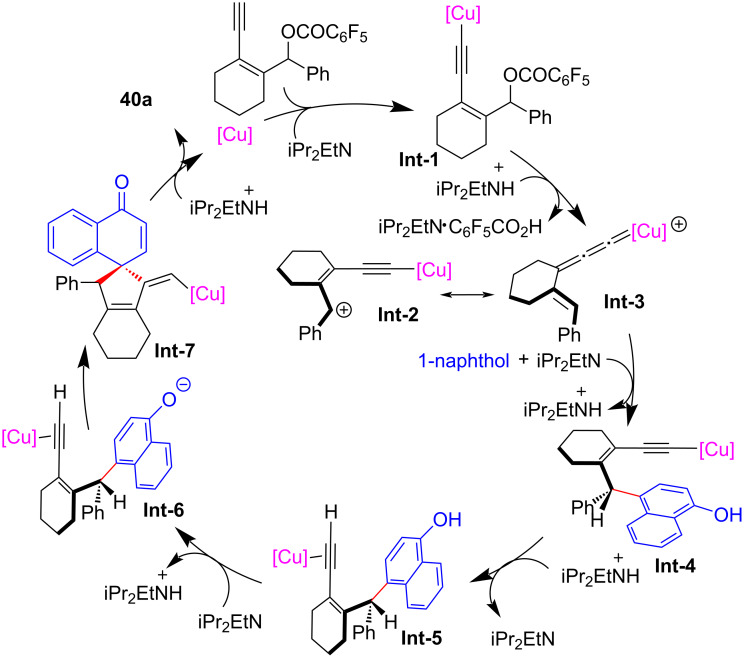
Postulated annulation mechanism.

Li, Yu and Liu et al. [78] achieved the asymmetric catalyzed dearomative [4 + 1] spiroannulation of nonfunctionalized 1-naphthol by applying a new ligand **L14**, leading to the rapid construction of differently substituted chiral spirocyclic enones **40a**–**j** with high yields and enantioselectivities (Scheme 41). It is worth noting that when the C4 position of 1-naphthol was occupied, the reaction occurred at the C2 position, resulting in the C2-dearomatized naphthalenone products **41a**–**d** with high efficiency (Scheme 41). In addition, electron-rich phenols or nonfunctionalized 2-naphthols could also be used as nucleophiles, providing the desired chiral spirocycles **43a**–**e** and **44a**–**e** in good yields with excellent ee values (Scheme 42). Preliminary mechanistic studies have ruled out the 1,3-sigmatropic shift, indicating that the reaction proceeds through a nucleophilic substitution–annulation process of a reactive π-extended copper-allenylidene intermediate (Scheme 43).

**Scheme 44:**
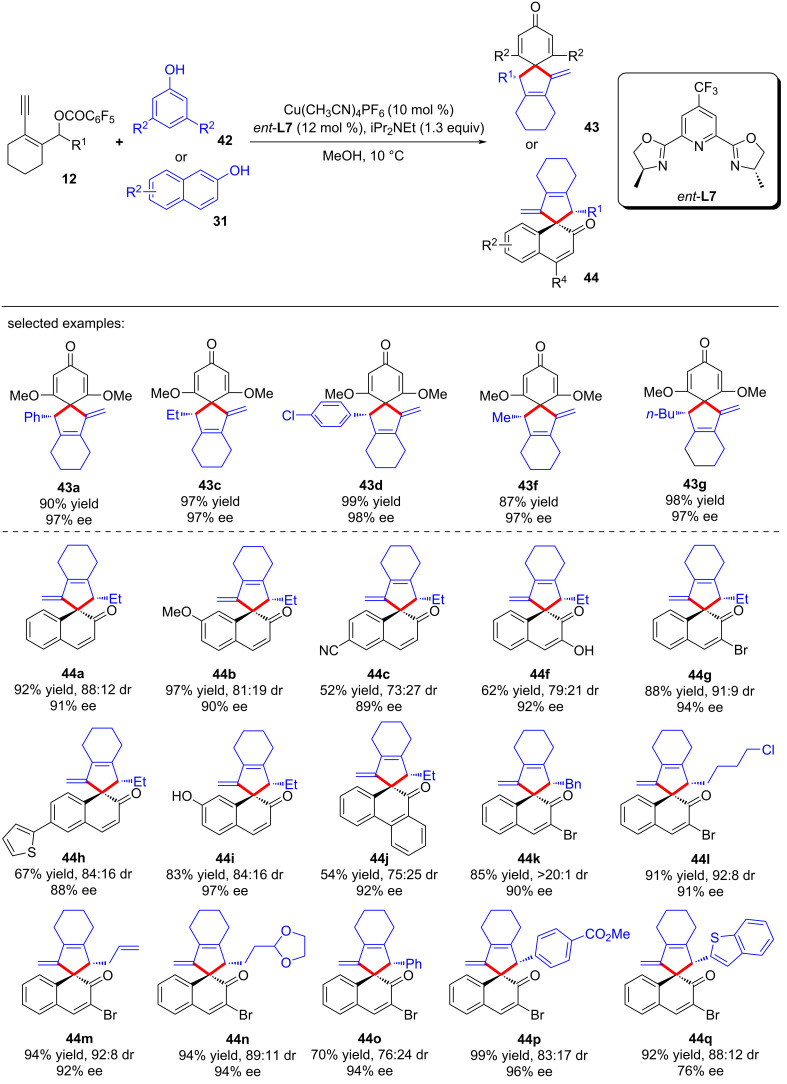
Dearomative annulation of phenols or 2-naphthols.

**Scheme 45:**
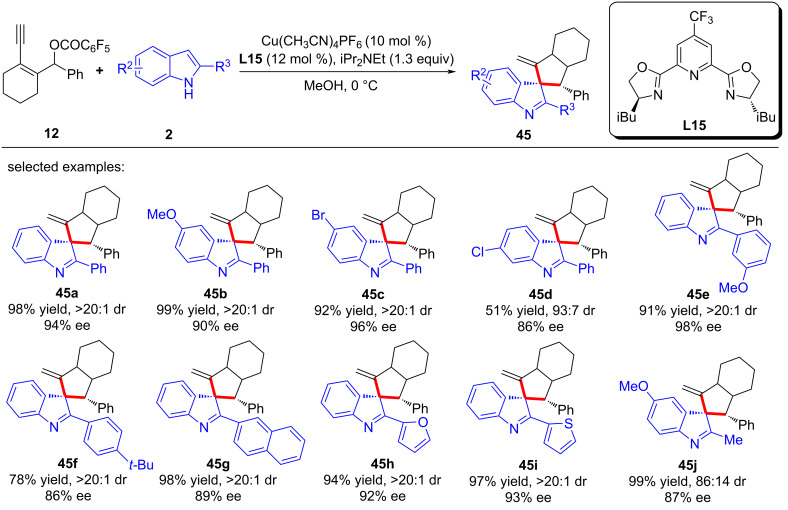
Dearomative annulation of indoles.

**Scheme 46:**
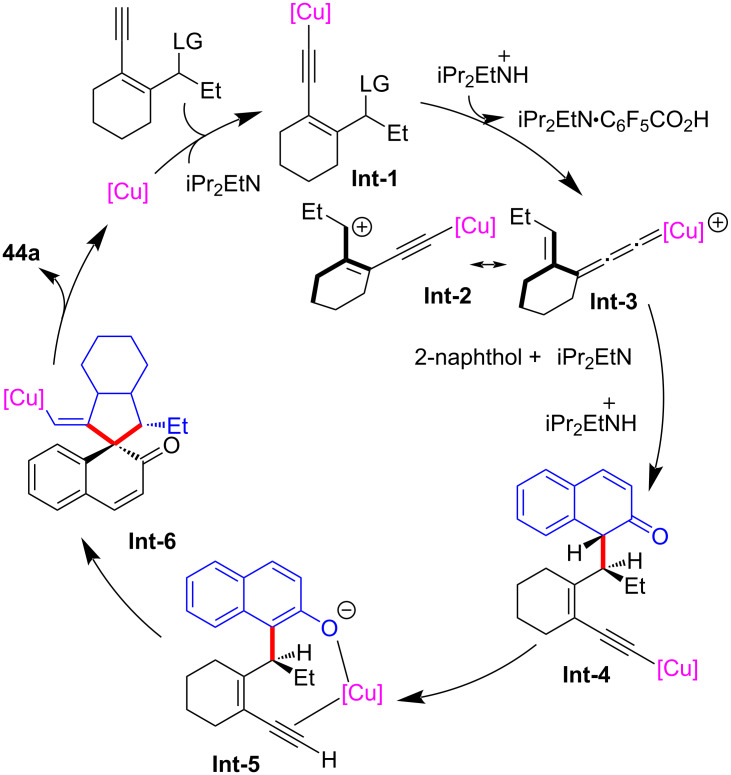
Postulated annulation step.

At the same time, Qi and Xu et al. [79] also realized the dearomative spiroannulation of 2-naphthols or electron-enriched phenols under mild conditions with excellent regioselectivities, enantioselectivities and diastereoselectivities (Scheme 44, **43a**–**g**, **44a**–**q**). In addition, the nucleophilic substitution–dearomative cyclization process between indoles and yne-allylic esters can also proceed smoothly, resulting in spiroindolenine derivatives with high yields and ee values (Scheme 45, **45a**–**j**). They also conducted mechanism studies and believed that the designed cyclic *cis*-yne-allylic esters are crucial for the progress of the reaction. The distal yne-allylic substitution is considered to be the determining step for the enantioselectivity, while the diastereoselectivity is mainly induced by the chiral alkylated naphthol intermediate in the second annulation step (Scheme 46).

**Scheme 47:**
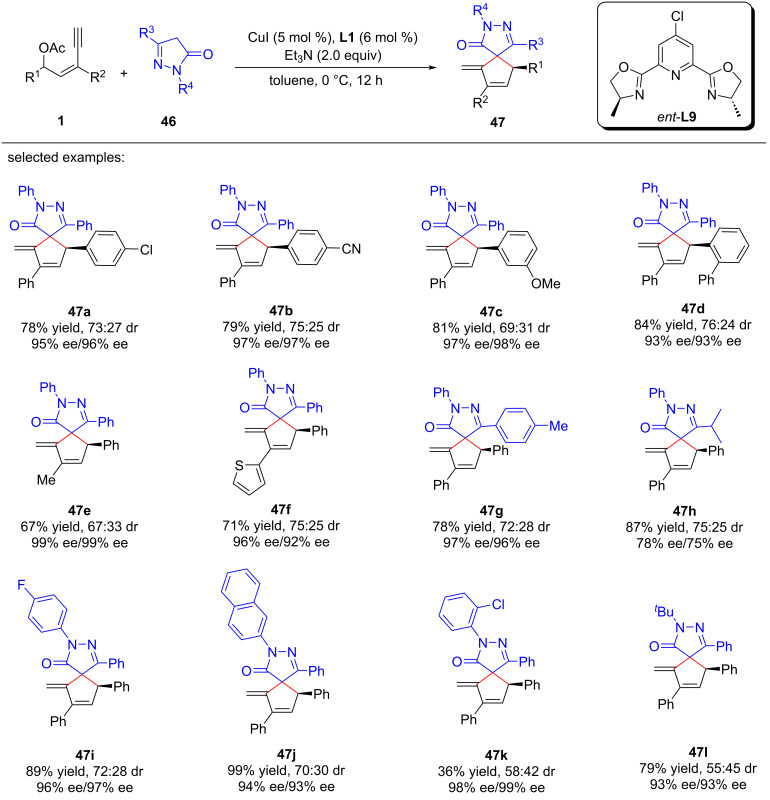
Asymmetric [4 + 1] cyclization of yne-allylic esters with pyrazolones.

**Scheme 48:**
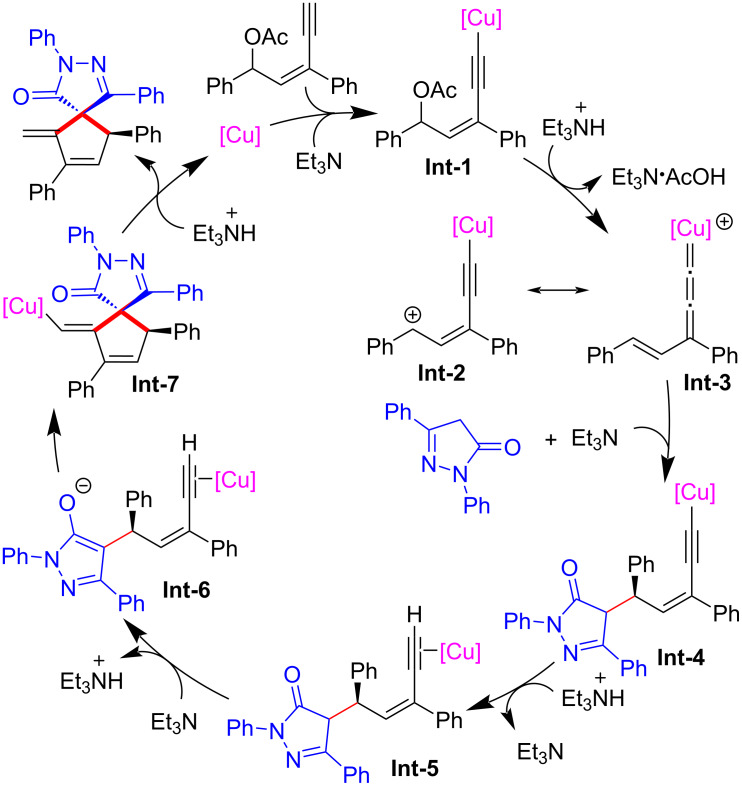
Proposed mechanism.

Xu et al. [80] realized asymmetric [4 + 1] cyclization of yne-allylic esters with pyrazolones. This catalytic strategy provided direct access to a range of chiral spiropyrazolones in good to high yields, displaying moderate to excellent enantiomeric excess (Scheme 47, **47a**–**l**). The method represents a novel approach for the synthesis of enantioenriched spirocyclic compounds with structural complexity. Through control experiment, they have proposed a reaction mechanism where the formation of copper vinyl allenylidene and Conia-ene reaction are pivotal steps in the process (Scheme 48).

**Scheme 49:**
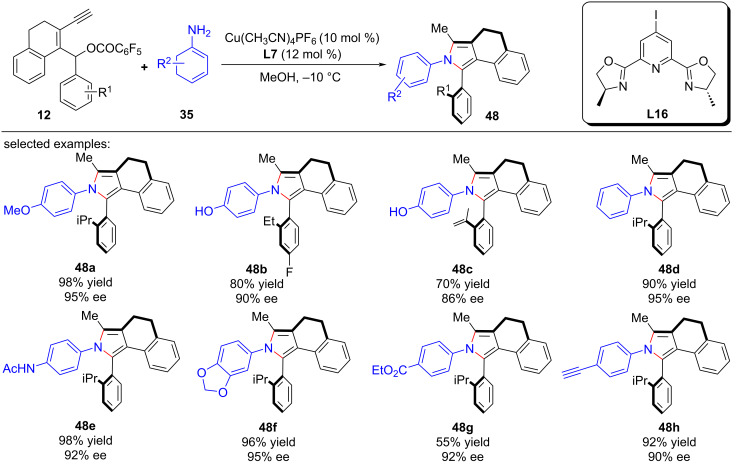
Construction of C–C axially chiral arylpyrroles.

**Scheme 50:**
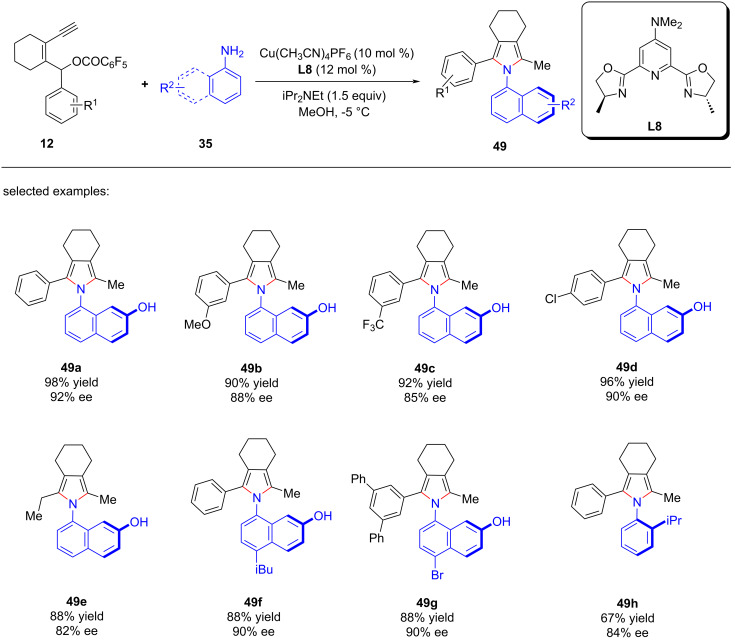
Construction of C–N axially chiral arylpyrroles.

**Scheme 51:**
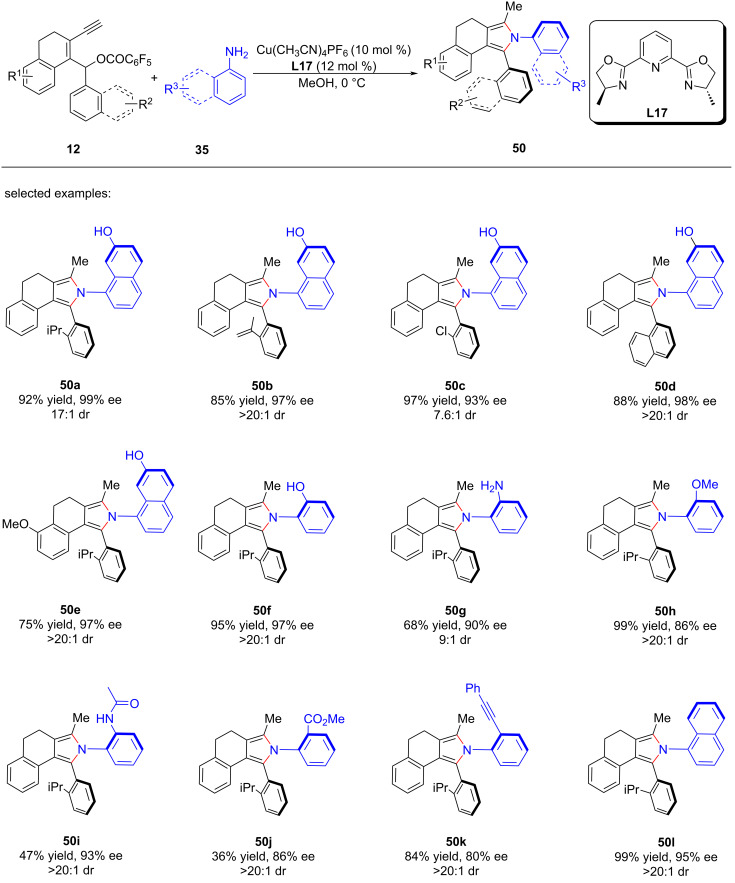
Construction of chiral arylpyrroles with 1,2-di-axial chirality.

**Scheme 52:**
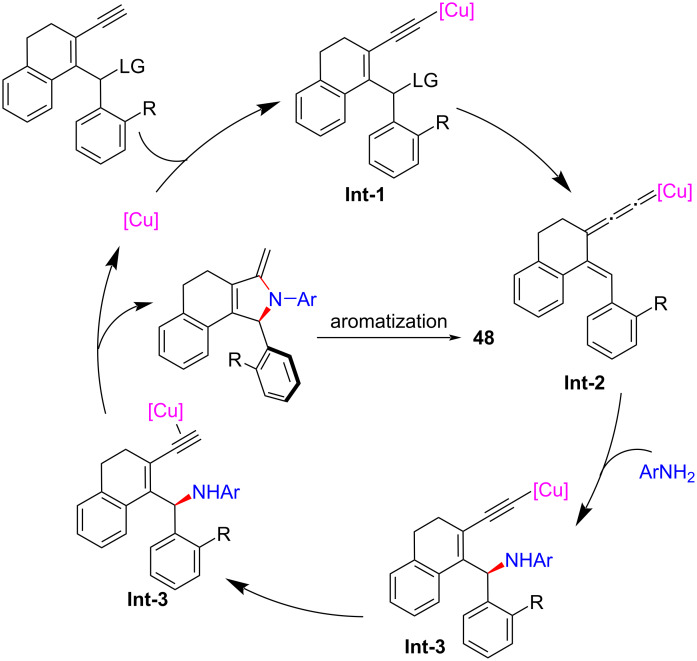
Proposed mechanism.

Crafting atropisomers, particularly for those with 1,2-diaxes, poses a formidable task owing to the intricate interplay of *ortho*-aryl steric hindrance. Recently, Xu et al. [81] presents a copper-catalyzed asymmetric [4 + 1] annulation strategy, utilizing remote stereocontrol substitution/annulation/aromatization to forge arylpyrroles with various C–C (Scheme 49, **48a**–**h**), C–N (Scheme 50, **49a**–**h**) or 1,2-di- (Scheme 51, **50a**–**l**) axial chirality in remarkable enantiopurities. Mechanistic studies and deuterium labeling experiments have revealed that the reaction proceeds in a stepwise manner without involving a 1,5-H migration process. Based on these findings, the authors have proposed a mechanism wherein the stereoselective aromatization serves as a pivotal step in the transfer of central chirality to axial chirality (Scheme 52).

**Scheme 53:**
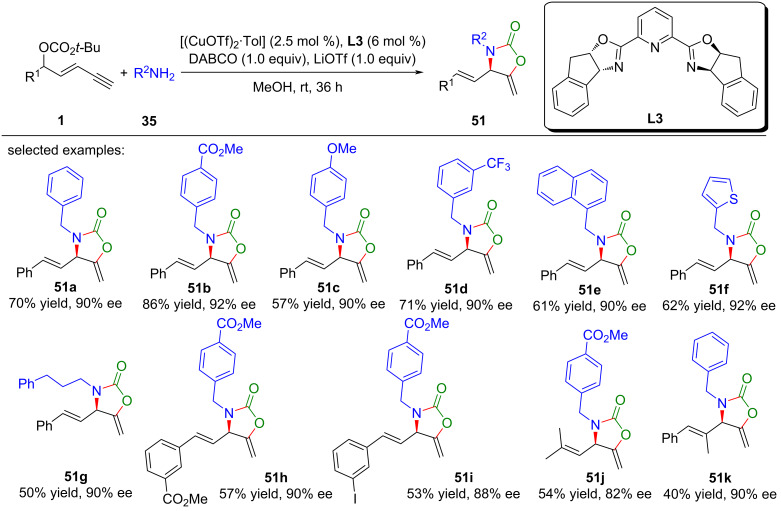
CO_2_ shuttling in yne-allylic substitution.

**Scheme 54:**
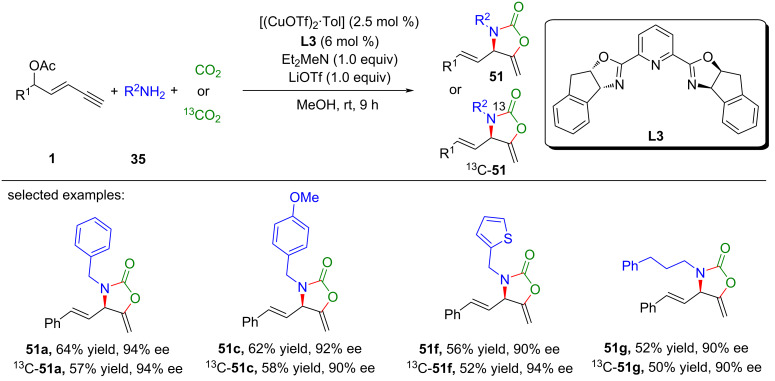
CO_2_ fixing in yne-allylic substitution.

**Scheme 55:**
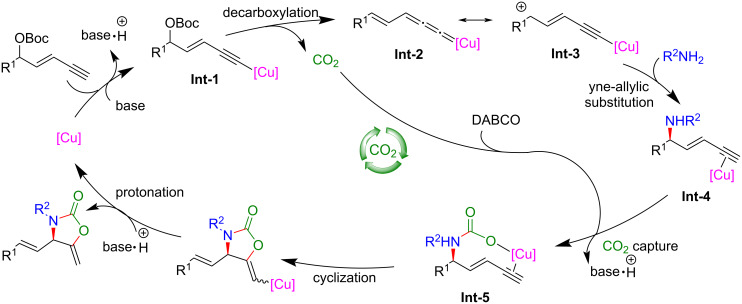
Proposed mechanism.

To harness the full potential of CO_2_ as a renewable and abundant carbon source, He et al. [82] proposed an innovative strategy that married asymmetric yne-allylic substitution with CO_2_ shuttling (Scheme 53, **51a**–**k**). Furthermore, they established a Cu-catalyzed asymmetric multicomponent reaction for yne-allylic substitution, seamlessly integrating ^13^C-labeled CO_2_ into enantiomerically pure products (Scheme 54, **51a**, **51c**, **51f**, **51g**). This methodology enabled the synthesis of diverse, high-value oxazolidinones with exceptional yields and enantioselectivities. This not only addresses the challenge of CO_2_ waste but also opens new avenues for the sustainable synthesis of complex molecules. Comprehensive mechanistic investigations underscored the pivotal role of DABCO in promoting CO_2_ capture and indicated that the carboxylative cyclization is the rate-limiting step in the overall pathway (Scheme 55).

## Conclusion

Since the first report in 2022, copper-catalyzed yne-allylic substitution has attracted intensive studies during the past two years. The protocol merges the features of both propargylic substitution and allylic substitution, but represents a new type of reaction mode, and greatly expands the scope of transition metal-catalyzed substitution reactions. Currently, yne-allylic substitutions affording 1,3- or 1,4-enynes, remote substitutions through dearomatization-rearomatization sequence, [4 + 1] and [3 + 2] annulations involving yne-allylic substitutions have been released. These studies have shown the huge potential of this protocol in affording diversified molecular scaffolds, and more studies will be expected to demonstrate the value of this reaction.

## Data Availability

Data sharing is not applicable as no new data was generated or analyzed in this study.
